# Supplementary material for: Transcriptome-Based WGCNA Analysis Reveals Regulated Metabolite Fluxes between Floral Color and Scent in Narcissus tazetta Flower
Source: Int J Mol Sci. 2021 Jul 31;22(15):8249. doi: 10.3390/ijms22158249 (PMC8348138; doi:10.3390/ijms22158249)
Supplement: Supplementary file 1 [file ijms-22-08249-s001.zip › ijms-1295118-supplementary.pdf]

Supplementary Table S1 Functional enrichment analysis of the transcription factor specific module of paracrown

Sample	module	GO_term	GO_ID	Items	Background Items	P_adj	Pvalue
C1	brown	Transcription	GO：0006351	15	399	0	0
		Regulation of transcription	GO：0006355	42	1472	0	0
		Radial pattern formation	GO：0009956	3	12	0.000372	2.42×10^{-6}
		Regulation of cellular process	GO：0050794	7	343	0.002058	1.34×10^{-5}
		Maintenance of protein location in nucleus	GO：0051457	2	3	0.002327	1.51×10^{-5}
		Defense response signaling pathway，resistance gene-independent	GO：0010204	3	49	0.029323	0.00019
		Response to stimulus	GO：0050896	6	372	0.031549	0.000205
		Defense response signaling pathway，resistance gene-dependent	GO：0009870	3	52	0.035001	0.000227
		Defense response to fungus	GO：0050832	5	245	0.037329	0.000242
C2	darkgrey	Response to lipid	GO：0033993	2	7	0.000385	5.42×10^{-6}
		Transcription	GO：0006351	4	399	0.003522	4.96×10^{-5}
		Regulation of transcription	GO：0006355	6	1472	0.00565	7.96×10^{-5}

		Positive regulation of transcription	GO：0045893	4	459	0.006056	8.53×10^{-5}
		Regulation of removal of superoxide radicals	GO：2000121	1	1	0.037003	0.000521
	tan	Regulation of transcription	GO：0006355	14	1472	7.72×10^{-11}	1.02×10^{-12}
		Transcription	GO：0006351	6	399	4.12×10^{-5}	5.42×10^{-7}
C3	black	Regulation of transcription	GO：0006355	25	1472	1.27×10^{-23}	1.34×10^{-25}
		Auxin-activated signaling pathway	GO：0009734	9	237	2.73×10^{-10}	2.87×10^{-12}
		Cellular response to osmotic stress	GO：0071470	3	15	4.51×10^{-5}	4.75×10^{-7}
		Response to fungus	GO：0009620	4	87	0.000202	2.13×10^{-6}
		Response to hormone	GO：0009725	4	115	0.000616	6.48×10^{-6}
		Post-translational protein modification	GO：0043687	4	116	0.000637	6.71×10^{-6}
		Transcription	GO：0006351	5	399	0.005551	5.84×10^{-5}
		Anthocyanin accumulation in tissues in response to UV light	GO：0043481	3	118	0.024564	0.000259
		Regulation of cellular process	GO：0050794	4	343	0.042503	0.000447
	darkturquoise	Regulation of transcription	GO：0006355	6	1472	0.00011	5.49×10^{-6}
		Transcription	GO：0006351	4	399	0.000175	8.76×10^{-6}
		Cellular biosynthetic process	GO：0044249	2	172	0.031877	0.001594
		Organonitrogen compound biosynthetic process	GO：1901566	1	6	0.04166	0.002083
C4	blue	Transcription	GO：0006351	20	399	0	0

		Regulation of transcription	GO：0006355	92	1472	1.06E-81	4.6×10^{-84}
		Cellular response to osmotic stress	GO：0071470	3	15	0.008145	3.53×10^{-5}
		Positive regulation of transcription	GO：0045893	10	459	0.008284	3.59×10^{-5}
		Response to o×ygen-containing compound	GO：1901700	5	93	0.012862	5.57×10^{-5}
		Response to hormone	GO：0009725	5	115	0.035172	0.000152
		Post-translational protein modification	GO：0043687	5	116	0.036628	0.000159
	green yellow	Regulation of transcription	GO：0006355	19	1472	6.22×10^{-17}	1.02×10^{-18}
		Actin filament bundle assembly	GO：0051017	2	41	0.03589	0.000588
P0	turquoise	Transcription	GO：0006351	23	399	0	0
		Regulation of transcription	GO：0006355	93	1472	1.2×10^{-81}	4.95×10^{-84}
		Determination of bilateral symmetry	GO：0009855	9	143	4.47×10^{-6}	1.85×10^{-8}
		Polarity specification of adaxial/abaxial axis	GO：0009944	8	104	5.87×10^{-6}	2.42×10^{-8}
		Primary shoot apical meristem specification	GO：0010072	7	115	0.000223	9.22×10^{-7}
		Post-translational protein modification	GO：0043687	7	116	0.000236	9.77×10^{-7}
		Integument development	GO：0080060	4	18	0.000272	1.12×10^{-6}
		Positive regulation of transcription	GO：0045893	12	459	0.000297	1.23×10^{-6}
		Auxin-activated signaling pathway	GO：0009734	8	237	0.003017	1.25×10^{-5}
		Radial pattern formation	GO：0009956	3	12	0.004531	1.87×10^{-5}

		Response to anoxia	GO：0034059	2	2	0.004803	1.98×10^{-5}
		Cellular response to osmotic stress	GO：0071470	3	15	0.009279	3.83×10^{-5}
		Flower morpHogenesis	GO：0048439	5	90	0.013162	5.44×10^{-5}
		Negative regulation of cell proliferation	GO：0008285	3	17	0.013777	5.69×10^{-5}
		Fruit dehiscence	GO：0010047	3	18	0.016478	6.81×10^{-5}
		Regulation of shoot system development	GO：0048831	3	21	0.026593	0.00011
		Leaf senescence	GO：0010150	4	58	0.032754	0.000135
		Negative regulation of biological process	GO：0048519	4	62	0.042471	0.000175
P1	midnight blue	Regulation of transcription	GO：0006355	12	1472	1.26×10^{-10}	5.03×10^{-12}
		Response to hormone	GO：0009725	3	115	0.000962	3.85×10^{-5}
		Integument development	GO：0080060	2	18	0.001185	4.74×10^{-5}
		Au×in-activated signaling pathway	GO：0009734	3	237	0.008154	0.000326
		Embryonic pattern specification	GO：0009880	2	99	0.036545	0.001462
		Transcription	GO：0006351	3	399	0.036825	0.001473
		Polarity specification of adaxial/abaxial axis	GO：0009944	2	104	0.04028	0.001611
		Primary shoot apical meristem specification	GO：0010072	2	115	0.049109	0.001964

P2	green	Regulation of transcription	GO：0006355	25	1472	1.05×10^{-22}	1.5×10^{-24}
		Transcription	GO：0006351	6	399	0.000374	5.34×10^{-6}
		Stomatal complex morpHogenesis	GO：0010103	4	139	0.001267	1.81×10^{-5}
		Trichome morpHogenesis	GO：0010090	4	257	0.013727	0.000196
P3	pink	Regulation of transcription	GO：0006355	18	1472	1.83×10^{-5}	3.05×10^{-17}
		Transcription	GO：0006351	5	399	0.001416	2.36×10^{-5}
		Response to hormone	GO：0009725	3	115	0.00832	0.000139
		Aromatic compound biosynthetic process	GO：0019438	2	25	0.013029	0.000217
		egulation of cellular process	GO：0050794	4	343	0.01316	0.000219
		Organic cyclic compound biosynthetic process	GO：1901362	2	33	0.02283	0.000381
P4	red	Regulation of transcription	GO：0006355	34	1472	3.78×10^{-35}	7.56×10^{-37}
		Transcription	GO：0006351	6	399	0.000561	1.12×10^{-5}

Supplementary Table S2 Transcription factors in the top 30% Hub genes of modules related to floral pigment and floral fragrance formation

Sample	Module	GeneID	Family
C3	yellow	c113566.graph_c0	AP2/ERF
	yellow	c115565.graph_c0	bZIP
	yellow	c111165/f1p0/3679	GARP
	yellow	c3312/f1p0/1393	HB
	yellow	c78506.graph_c0	HB
	yellow	c111781.graph_c0	LOB
	yellow	c109881.graph_c0	LOB
	yellow	c12422/f2p1/1484	MYB
	yellow	c122194.graph_c0	MYB
	yellow	c110400.graph_c0	MYB
	yellow	c123531.graph_c1	NAC
	yellow	c26651/f1p0/1318	PLATZ
	yellow	c50868/f1p1/2479	WD40
	yellow	c88692.graph_c0	ZF
P1	bisque4	c115272.graph_c0	AP2/ERF
	bisque4	c38849.graph_c0	AP2/ERF
	bisque4	c114283.graph_c0	AP2/ERF
	bisque4	c112446.graph_c0	AP2/ERF
	bisque4	c101703.graph_c0	B3
	bisque4	c17677/f1p0/1250	BBR
	bisque4	c45912.graph_c0	bHLH
	bisque4	c119890.graph_c0	bHLH
	bisque4	c20405/f1p0/1640	bZIP
	bisque4	c3099/f1p8/1864	bZIP
	bisque4	c108797.graph_c0	bZIP
	bisque4	c38896/f1p1/1760	C2C2
	bisque4	c17081/f1p0/1503	C2C2
	bisque4	c106647.graph_c0	C2C2
	bisque4	c103341.graph_c0	C2C2
	bisque4	c119142.graph_c0	C2H2
	bisque4	c116644.graph_c1	C2H2
	bisque4	c114645.graph_c0	C2H2
	bisque4	c120999.graph_c0	C3H
	bisque4	c114457.graph_c0	C3H
	bisque4	c23113/f1p1/1494	MYB
	bisque4	c9225/f4p2/1499	MYB
	bisque4	c125126.graph_c0	MYB
	bisque4	c106256.graph_c0	MYB
	bisque4	c44176.graph_c0	MYB
	bisque4	c103435.graph_c0	NAC

	bisque4	c110425.graph_c0	NAC
	bisque4	c3131/f1p0/1551	NF
	bisque4	c114160.graph_c0	NF
	bisque4	c108405.graph_c0	OFP
	bisque4	c107896/f1p0/3456	S1Fa
	bisque4	c112561.graph_c0	SBP
	bisque4	c95432.graph_c0	SBP
	bisque4	c112166.graph_c0	SBP
	bisque4	c24298/f1p0/1887	TCP
	bisque4	c120592.graph_c0	ZF
P3	brown4	c47831/f1p0/2554	AP2/ERF
	brown4	c112584.graph_c0	AP2/ERF
	brown4	c128472.graph_c0	AP2/ERF
	brown4	c89954.graph_c0	AP2/ERF
	brown4	c116432.graph_c0	AP2/ERF
	brown4	c44698.graph_c0	B3
	brown4	c28841/f1p0/2147	bHLH
	brown4	c68212/f1p1/2519	bHLH
	brown4	c104883/f1p0/3411	bHLH
	brown4	c18545/f1p0/1492	bHLH
	brown4	c119116.graph_c0	bHLH
	brown4	c99947.graph_c0	bHLH
	brown4	c78553.graph_c0	bHLH
	brown4	c4999/f1p0/1943	bZIP
	brown4	c4492/f1p1/1394	bZIP
	brown4	c18042/f1p0/1260	bZIP
	brown4	c38140/f1p1/1562	bZIP
	brown4	c106708.graph_c0	bZIP
	brown4	c100911.graph_c0	bZIP
	brown4	c120609.graph_c0	bZIP
	brown4	c114078.graph_c0	bZIP
	brown4	c111846.graph_c0	bZIP
	brown4	c106657.graph_c0	C2C2
	brown4	c111269.graph_c0	C2H2
	brown4	c109204.graph_c0	C2H2
	brown4	c14695/f1p1/1708	E2F
	brown4	c18739/f1p0/1744	GARP
	brown4	c117164.graph_c0	GARP
	brown4	c26500/f1p0/2093	GRAS
	brown4	c14406/f1p0/1538	HB
	brown4	c62685/f1p4/2588	HB
	brown4	c24359/f1p0/1573	HB
	brown4	c70931/f1p0/2659	HB
	brown4	c105951.graph_c0	HB

	brown4	c123323.graph_c0	HB
	brown4	c110700/f1p0/3535	MYB
	brown4	c16798/f1p0/1420	MYB
	brown4	c12422/f2p1/1484	MYB
	brown4	c115475.graph_c0	MYB
	brown4	c95617.graph_c1	MYB
	brown4	c115335.graph_c0	MYB
	brown4	c103885.graph_c0	MYB
	brown4	c48011/f1p0/2620	NAC
	brown4	c13238/f2p0/1653	NAC
	brown4	c3490/f1p3/1647	NAC
	brown4	c18463/f1p0/1184	NAC
	brown4	c120112.graph_c1	NAC
	brown4	c120475.graph_c1	NAC
	brown4	c120475.graph_c0	NAC
	brown4	c116889.graph_c0	NF
	brown4	c117231.graph_c0	OFP
	brown4	c99833.graph_c0	OFP
	brown4	c26651/f1p0/1318	PLATZ
	brown4	c86440.graph_c0	PLATZ
	brown4	c113435.graph_c0	Tify
	brown4	c10900/f2p0/1623	ULT
	brown4	c4425/f1p1/1606	WD40
	brown4	c8058/f2p0/1919	WRKY
	brown4	c100391.graph_c0	WRKY
	brown4	c109107/f1p0/3571	ZF
	brown4	c104859.graph_c0	ZF
P4	coral1	c2036/f3p0/1600	AP2/ERF
	coral1	c47831/f1p0/2554	AP2/ERF
	coral1	c11421/f2p1/2051	AP2/ERF
	coral1	c116432.graph_c0	AP2/ERF
	coral1	c120995.graph_c0	AP2/ERF
	coral1	c102297.graph_c0	AP2/ERF
	coral1	c104883/f1p0/3411	bHLH
	coral1	c18545/f1p0/1492	bHLH
	coral1	c13244/f2p2/1422	bHLH
	coral1	c121995.graph_c0	bHLH
	coral1	c18042/f1p0/1260	bZIP
	coral1	c38140/f1p1/1562	bZIP
	coral1	c4492/f1p1/1394	bZIP
	coral1	c115833.graph_c0	bZIP
	coral1	c120609.graph_c0	bZIP
	coral1	c105952.graph_c0	bZIP
	coral1	c21292/f1p0/1595	C2C2

coral1	c114771.graph_c0	C2C2
coral1	c120161.graph_c1	C2H2
coral1	c109204.graph_c0	C2H2
coral1	c122144.graph_c0	DBP
coral1	c14695/f1p1/1708	E2F
coral1	c117164.graph_c0	GARP
coral1	c106358.graph_c1	GARP
coral1	c26500/f1p0/2093	GRAS
coral1	c24359/f1p0/1573	HB
coral1	c23536/f1p0/1779	HB
coral1	c14406/f1p0/1538	HB
coral1	c17089/f1p0/1242	HB
coral1	c70931/f1p0/2659	HB
coral1	c123323.graph_c0	HB
coral1	c118634.graph_c0	HB
coral1	c104894.graph_c1	HB
coral1	c105951.graph_c0	HB
coral1	c82867/f1p1/4332	HSF
coral1	c98286.graph_c0	LOB
coral1	c8658/f2p1/1443	MYB
coral1	c20734/f1p1/1464	MYB
coral1	c23880/f1p0/1508	MYB
coral1	c55262.graph_c0	MYB
coral1	c115335.graph_c0	MYB
coral1	c98415.graph_c0	MYB
coral1	c107671.graph_c0	MYB
coral1	c3490/f1p3/1647	NAC
coral1	c18463/f1p0/1184	NAC
coral1	c39424/f1p0/1351	NAC
coral1	c7615/f1p3/1491	NAC
coral1	c48011/f1p0/2620	NAC
coral1	c120475.graph_c0	NAC
coral1	c120112.graph_c1	NAC
coral1	c120475.graph_c1	NAC
coral1	c120933.graph_c0	NAC
coral1	c108608.graph_c0	NF
coral1	c92926.graph_c0	Trihelix
coral1	c10900/f2p0/1623	ULT
coral1	c8058/f2p0/1919	WRKY
coral1	c100391.graph_c0	WRKY
coral1	c87064.graph_c0	WRKY
coral1	c119047.graph_c1	WRKY
coral1	c124558.graph_c1	WRKY

Supplementary Figure S1-S5

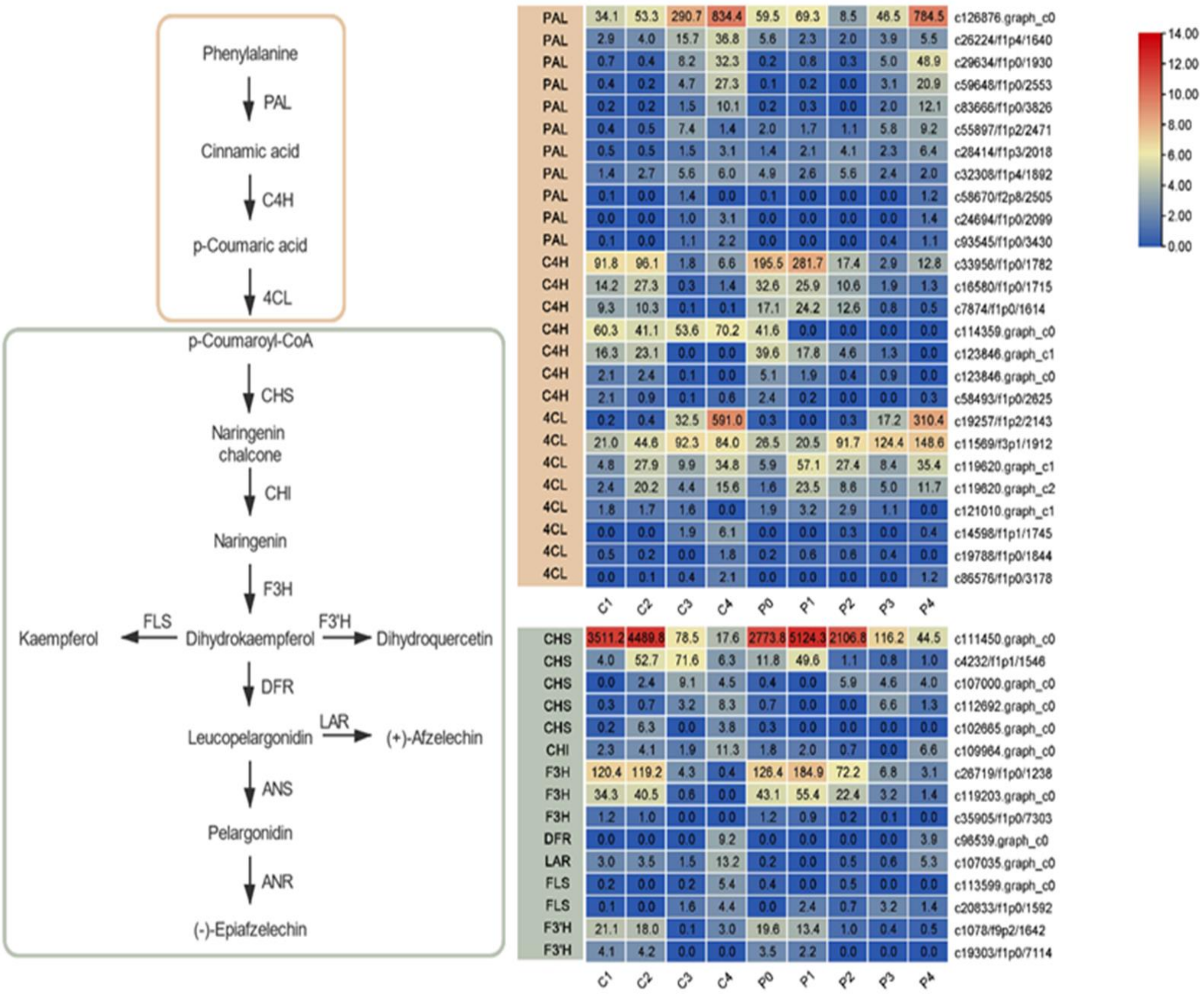

Supplementary Figure S1. The expression patterns analysis of genes related to flavonoids metabolic pathway in petals and coronas of narcissus

The red box in the left panel represents the phenylpropanoid biosynthesis pathway, and the gray box represents the flavonoid biosynthesis pathway. The heat map shows the expression patterns of differentially expressed genes related to flavonoids metabolic pathway in petals and coronas at different periods. Enzyme names are shown on the left side of the heat map. Enzymes in the phenylpropanoid biosynthesis are shown in red background and enzymes in the flavonoid biosynthesis pathway are shown in gray. The FPKM values of genes were labeled on the heat map.

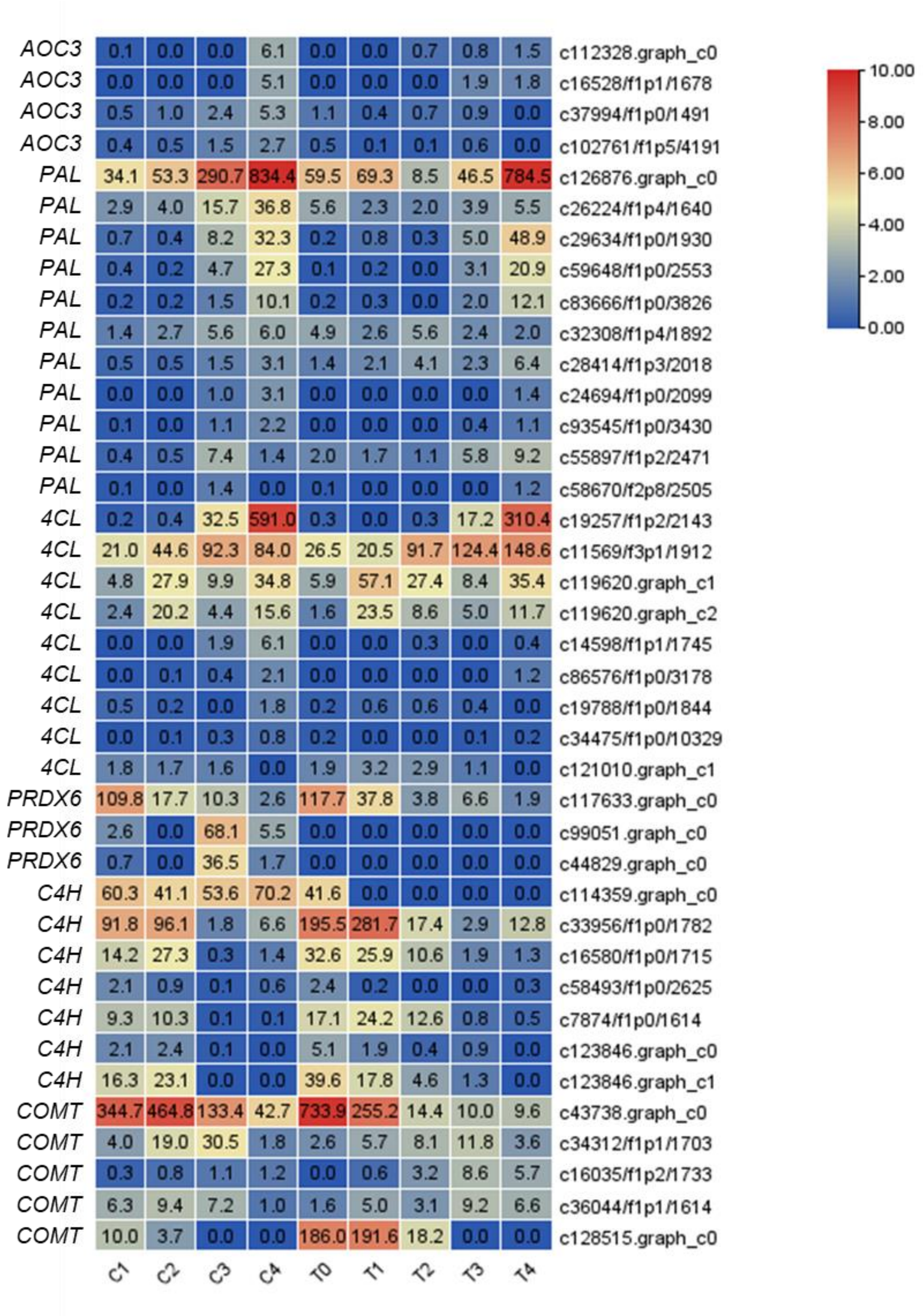

Supplementary Figure S2 The expression patterns analysis of genes related to phenylpropanoid biosynthesis in tepals and coronas of narcissus

The red box in the left panel represents the phenylpropanoid biosynthesis pathway, and the gray box represents the flavonoid biosynthesis pathway. The heat map shows the expression patterns of differentially expressed genes related to phenylpropanoid biosynthesis pathway in petals and coronas at different periods. Enzyme names are shown on the left side of the heat map. The FPKM values of genes were labeled on the heat map.

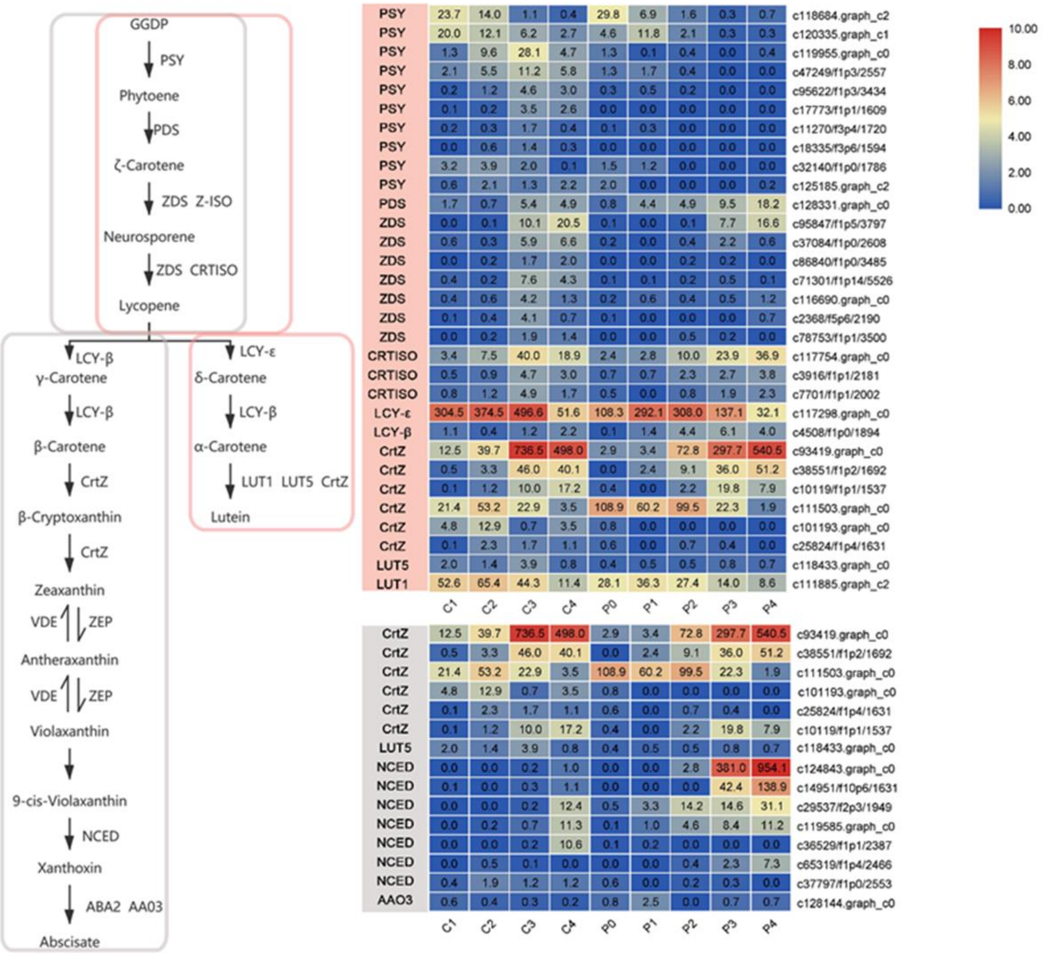

Supplementary Figure S3. The expression patterns analysis of genes related to carotenoids metabolic pathway in petals and coronas of Chinese narcissus

The pathway is divided intoβ-carotene and lutein synthesis sub-pathways based on the functions of unigenes. The red box in the left panel represents the carotenoid synthesis pathway, and the gray box represents the carotenoid degradation pathway. The heat map shows the expression patterns of differentially expressed genes related to carotenoids metabolic pathway in petals and coronas at different periods. Enzyme names are shown on the left side of the heat map. Enzymes in the carotenoid synthesis pathway are shown in red background and enzymes in the degradation pathway are shown in gray. The FPKM values of genes were labeled on the heat map.

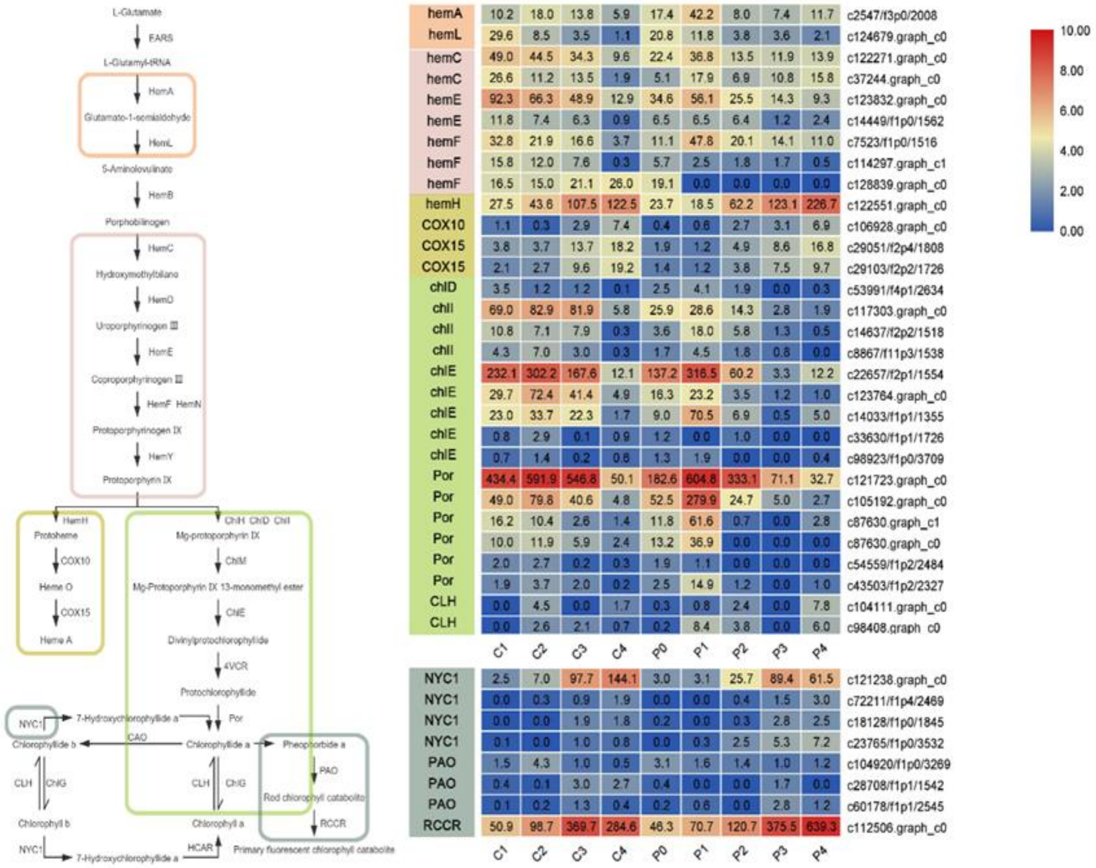

Supplementary Figure S4 The expression patterns analysis of genes related to chlorophyll metabolic pathway in petals and coronas of Chinese narcissus

The pathway is divided into five associated parts (ALA formation, Proto IX formation, heme formation, chlorophyll formation, and chlorophyll breakdown) based on the functions of unigenes. The heat map shows the expression patterns of differentially expressed genes related to chlorophyll metabolic pathway in petals and coronas at different periods. The names of enzymes were shown on the left side of the heat map and different background colors represented different sub-pathways. From top to bottom, there were ALA formation, Proto IX, heme formation, chlorophyll formation and chlorophyll degradation pathway related enzymes. The FPKM values of genes were labeled on the heat map.

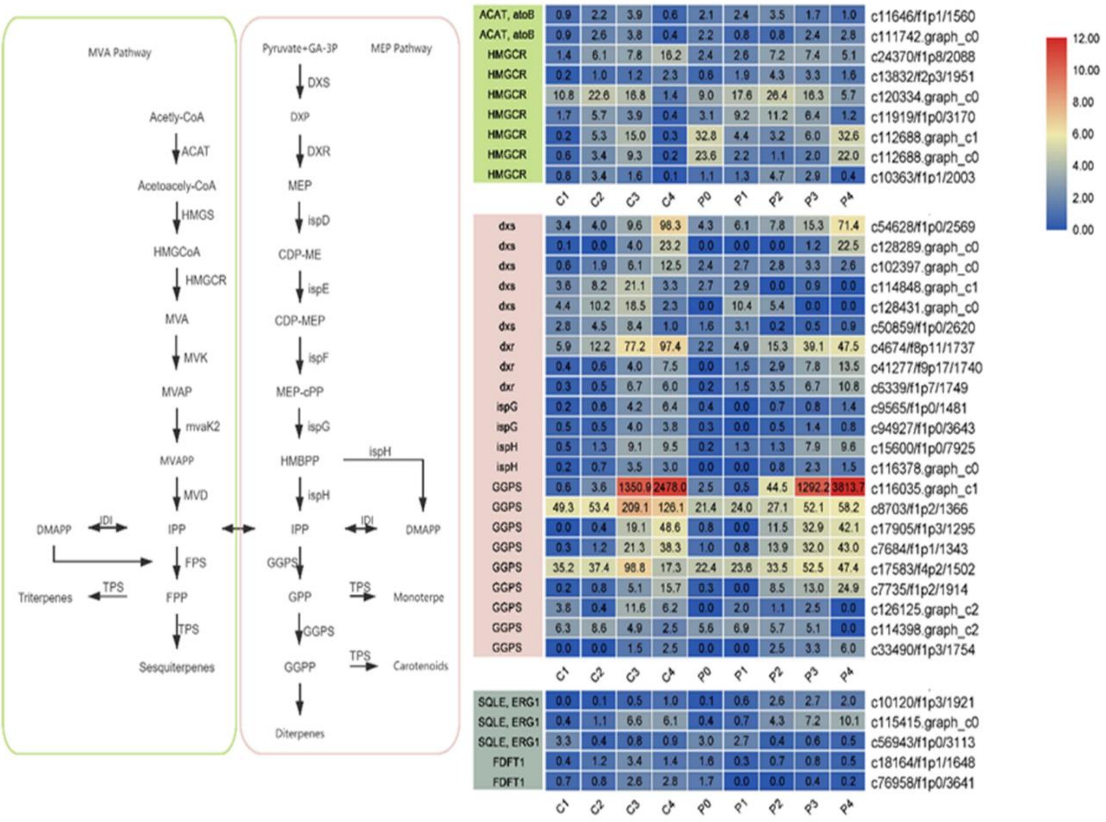

Supplementary Figure S5. The expression patterns analysis of genes related to terpenoid metabolic pathway in petals and coronas of narcissus

The pathway is divided into MVA pathway and MEP pathway based on the functions of unigenes. The green box in the left panel represents the MVA synthesis pathway, and the pink box represents the MEP synthesis pathway. The heat map shows the expression patterns of differentially expressed genes related to terpenoid metabolic pathway in petals and coronas at different periods. Enzyme names are shown on the left side of the heat map. Enzymes in MVA pathway are shown in red background, enzymes in the MEP pathway are shown in pink background, and enzymes related to monoterpenes and sesquiterpenes are in grey-green. The FPKM values of genes were labeled on the heat map.

(A)

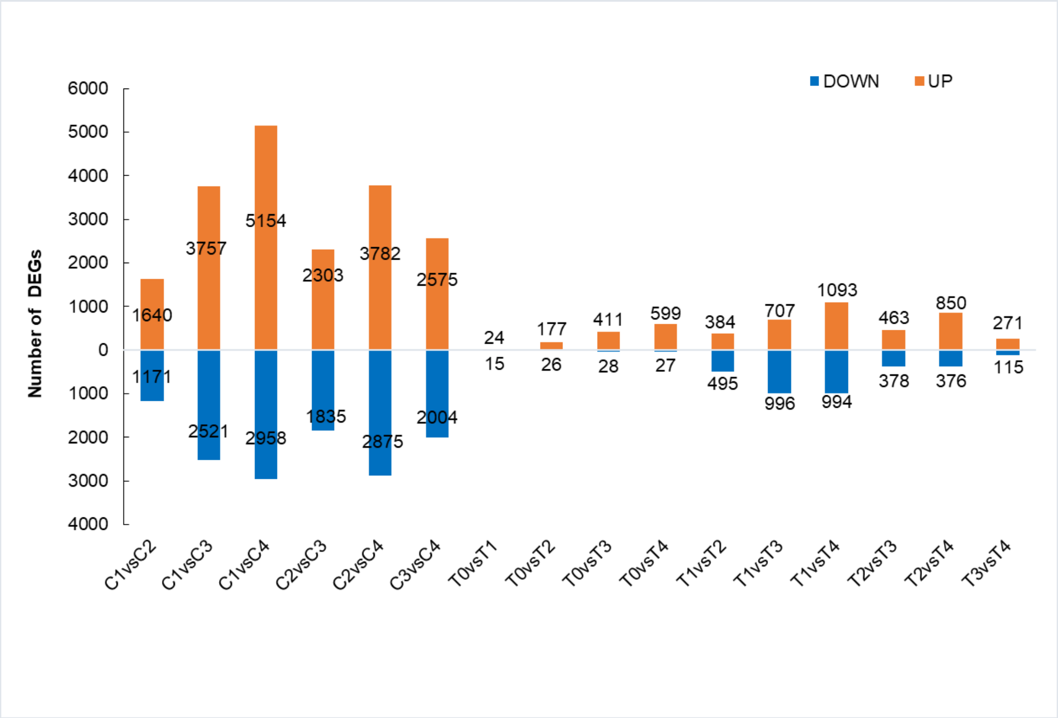

(B)

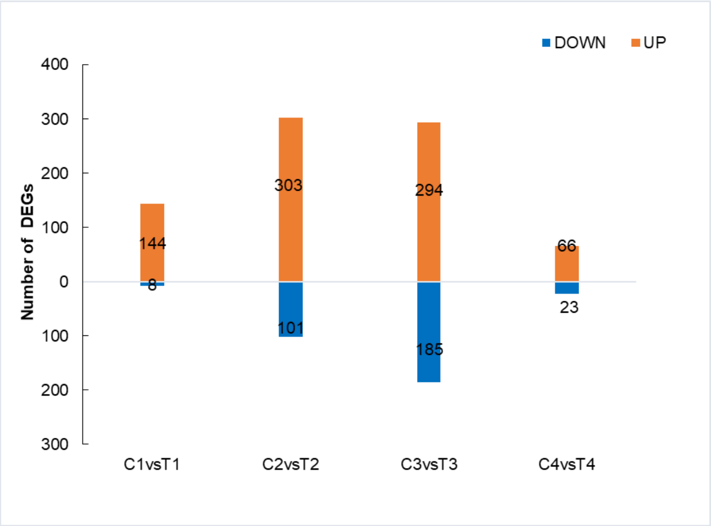

Supplementary Figure S6. Analysis of differentially expressed transcription factor genes in the tepal and corona of narcissus. (A) Number of up and down-regulated genes in petals or coronas of narcissus at different periods; (B) Number of up and down-regulated genes between petals and corollas of narcissus at the same periods.
